# Work disability status following routine mental health treatment: a Norwegian registry-based cohort study

**DOI:** 10.1186/s12913-025-12856-w

**Published:** 2025-06-02

**Authors:** Jakob Lundqvist, Martin Schevik Lindberg, Martin Brattmyr, Audun Havnen, Lene Aasdahl, Stian Solem, Odin Hjemdal

**Affiliations:** 1 Department of Psychology, Norwegian University of Science and Technology (NTNU), Trondheim, Norway; 2 Health and Welfare, Trondheim Municipality, Trondheim, Norway; 3 Division of Psychiatry, Nidaros Community Mental Health Centre, St. Olavs University Hospital, Trondheim, Norway; 4 Department of Public Health and Nursing, Norwegian University of Science and Technology (NTNU), Trondheim, Norway; 5 Unicare Helsefort Rehabilitation Center, Rissa, Trondheim, Norway; 6 Diakonhjemmet Hospital, Oslo, Norway

**Keywords:** Sick leave, Absenteeism, Mental disorders, Treatment as usual, Work disability

## Abstract

**Objectives:**

Sickness absence due to mental disorders is increasing in many high-income countries, yet the impact of routine mental health treatment on work disability outcomes remains unclear. This study examined trajectories of work disability (working, partly or full sick leave, Work Assessment Allowance, or disability pension) before and after routine mental health care and identified factors associated with work disability status.

**Methods:**

A prospective cohort of 2,609 adult outpatients with mild to severe mental disorders receiving routine mental health treatment in Norwegian community or specialist services was followed. Registry data tracked work disability status one year before and after treatment. Group-based trajectory modelling, sequence clustering, and multinomial logistic regression were used.

**Results:**

Work disability increased sharply in the year prior to treatment, peaking at 38% at treatment start. Although a modest decline in absenteeism followed, long-term medical benefits of Work Assessment Allowance steadily rose from 7% pre-treatment to 18% post, a 152% increase, contributing to sustained levels of absenteeism after start of treatment. Five trajectories were identified, with 46% maintaining stable work ability. Approximately 30% of patients, corresponding to clusters 3 and 4, experienced reduced sick leave with benefit transition to long-term medical benefits, while 7% were permanently on disability pension. Older age, female sex, and treatment in specialist services were associated with higher work disability, while community services targeting mild to moderate conditions were linked to better work ability outcomes.

**Conclusions:**

Work disability increased sharply before treatment and remained persistently high throughout the following year, forming a plateau across care levels. Although nearly half of the patients maintained stable work ability, the findings indicate that routine mental health care, especially specialist services, may have limited effectiveness in preventing long-term work disability. A substantial proportion transitioned into long-term medical benefits, highlighting the urgent need for timely interventions that integrate work ability as a core treatment objective and apply targeted interventions to enhance it.

**Supplementary Information:**

The online version contains supplementary material available at 10.1186/s12913-025-12856-w.

## Introduction

Mental disorders are one of the leading causes of work disability globally [1], leading to significant financial, social, and personal consequences [2, 3]. Norway has, by far, the highest sick leave and disability benefit rates across OECD countries, with 7% of the workforce on daily absence compared to the OECD average of around 3% [4, 5]. Mental disorders are the second most common cause of medically certified sick leave days in Norway, following cardiovascular diseases, and have the longest average duration [6]. However, the true prevalence of mental health-related absenteeism is likely higher due to underreporting of psychiatric causes [7].

In recent years, Norway has seen a sharp increase in long-term temporary medical benefits, labelled Work Assessment Allowance (WAA), and permanent disability pensions due to mental disorders, particularly among young adults [8, 9]. Although evidence-based treatments are available for many mental disorders [10], their impact on functional outcomes, such as work disability, remains less clear [11, 12]. Work disability is often considered a secondary outcome in both research and routine mental healthcare [11–13].

Research on the effectiveness of psychological treatments in improving work ability remains inconclusive, with mixed findings across studies. While some studies indicate that psychotherapy, such as cognitive–behavioural therapy (CBT), had some or a small positive effect on work disability [13–15], others show no significant advantage [16, 17]. Whereas work-focused interventions alone have demonstrated limited work ability improvements, the combination with psychological interventions may shorten the time to the first day of returning to work [18–20].

However, findings from studies using treatment as usual (TAU) as a control condition can be difficult to interpret. Although TAU is commonly used as a control condition in clinical trials, it is rarely defined, described and investigated in detail [21–23]. While experimental interventions typically receive primary attention, TAU often reflects unsystematic care rather than a clearly specified clinical intervention, thereby limiting the interpretability and generalisability of findings to routine practice [24, 25]. It is crucial to understand not only the effect of specific treatment methods on work disability in specialised settings, but also the outcome of the treatment most patients receive [1]. In routine care, TAU typically refers to a broad range of therapeutic methods provided to patients with diverse backgrounds by therapists with regular caseloads [26]. Yet, few studies have examined how work disability evolves before and after treatment in naturalistic settings.

A systematic review highlighted that few studies have addressed changes in work disability following the start of routine mental health care [27]. Among those included, the Swedish primary healthcare study reported a 24% relative reduction in sick leave after six months (from 55 to 42% ), though pre-treatment data and treatment duration were unspecified [28]. Another Swedish trial found a decrease from 61 net days to 17 sick leave days after nine months, with no significant differences between TAU and specific treatment methods; patients had an average of 7 sessions, of which 4 were with a psychologist [29]. A third included study from Sweden reported that only 3.4% improved their sick leave status, without details of sick leave days or treatment duration [30]. Additionally, a Finnish register-based study established a 20% reduction in sickness absence after 4 years, with a mean treatment duration of three years. However, absenteeism remained higher three years post-treatment compared to levels three years before the start [31]. Similarly, a Norwegian outpatient study focused on outpatients without prior long-term medical benefits, found that while sick leave decreased post-treatment, long-term improvements were limited. Notably, 25% received long-term medical benefits within one year, with those initially on full sick leave at treatment start appearing particularly vulnerable to long-term medical benefits, regardless of symptom severity [32].

Despite increasing levels of sickness absence due to mental disorders, work disability patterns before and after treatment remain unclear, as few studies present such data [1, 27, 33]. Understanding different patterns following mental health treatment is crucial for improving return-to-work interventions. While most existing studies focus on symptom reduction and outcomes following start of treatment, few have tracked changes in work disability across both the pre- and post-treatment periods. As a result, critical questions remain regarding whether, and at what point, work disability occurs across different clinical settings (primary and specialist care).

To address this gap, this study aimed to identify trajectories of work disability among patients receiving routine mental health care, irrespective of symptom severity or prior benefit use, and to examine sociodemographic and clinical predictors of successful or unsuccessful work outcomes. Based on previous research [31, 32], we hypothesised that work disability would increase prior to treatment, decrease following treatment, and be accompanied by a potential rise in long-term medical benefit.

## Methods

### Study design and setting

This prospective cohort study includes data from adults receiving routine outpatient mental health care in a large Norwegian city. Patients were recruited from two levels of care: Community mental health services and Specialist mental health services. Patients starting Specialist mental health treatment between February 2020 and February 2022, and those starting Community mental health services between September 2020 and October 2022, were included. This study period overlapped with the COVID-19 pandemic, contributing to an overall rise in sick leave, likely due to respiratory infections, while the prevalence of mental disorders remained stable in this region [34].

### Participants

Patients eligible for treatment within Specialist mental health services, or who sought help through Community mental health services, received an automatic text message some days before the start of treatment, containing an invitation to participate and a link to a web-based portal with detailed project information. Informed consent was obtained electronically through the portal. Patients referred to Specialist mental health services with certain disorders, such as obsessive-compulsive disorder, schizophrenia, and substance abuse issues, were not included as they were provided treatment in other specialised units. Patients above 67 years (*n =* 26) were excluded due to retirement eligibility. The final sample consisted of 2,609 outpatients.

### Data sources and measurement

Data on medical benefits were obtained from the National Social Security System Registry, managed by the Norwegian Labour and Welfare Administration (NAV). This registry records all individuals in Norway receiving any type of medical benefits, identified by social security number. For each participant, we retrieved data on the three different medical benefits existing in Norway: sick leave, WAA, and disability pension. Data included benefit start/end dates, grading (level of benefits if graded), and associated medical diagnoses classified primarily according to the International Classification of Primary Care, 2nd edition (ICPC-2) [35]. Participants’ ages and sex were retrieved from their social security numbers.

### Context: the Norwegian sickness benefit system

In Norway, the certification of sickness benefits and the provision of psychological treatment are typically managed separately, with GPs usually responsible for medical benefit certification, NAV providing the benefit, and mental health services providing psychological treatment. These stakeholders often operate sequentially, with limited coordination of efforts [36]. *Sick leave benefits (Short-term*,* Time-limited: Partial or Full)* provide 100% coverage of medically certified sick leave, whether partial or full, for a maximum of 52 weeks. Eligibility requires at least four weeks of Norwegian employment and membership in the Norwegian National Insurance Scheme (with some provisions for EU/EEA citizens) [4]. *Work Assessment Allowance (WAA) (Temporary, Long-term)* is an extended long-term sick leave benefit that individuals may apply for after 52 weeks of sick leave, typically providing coverage for 66% of their previous income for up to three years. The primary goal of WAA is to support individuals in transitioning back to suitable work through treatments and work-focused initiatives, such as labour market programmes and occupational rehabilitation [4]. *Disability pension* provides long-term income support for those permanently unable to work due to disorders or disability. It sums 66% of the average income from their three highest income-bringing years out of five before sick leave. It is typically granted after medical and vocational assessments, often following the exhaustion of other medical benefits [4].

### Study context: routine treatment interventions

Participants received routine care within the Norwegian public mental health care system, typically by general practitioners (GP), psychologists, psychiatrists, psychiatric nurses, or social workers. The Norwegian mental health system follows a stratified care model, ensuring access to treatment based on the severity and complexity of conditions. Community mental health services (primary healthcare), including GPs, are managed by municipalities, while Specialist mental health services provide secondary care.

Community mental health services have two main target groups: *Community—mild to moderate* (Subsample 1, *n*_*1*_ = 419) offer early interventions for individuals seeking treatment for recently developed mild to moderate conditions. Interventions include CBT-based approaches like guided self-help, psycho-educative courses, group therapy, and individual therapy. Additionally, there are interventions aimed at grief, trauma, and support for those experiencing domestic violence. Patients in this subsample received a median of 6 sessions (*M* = 7.06, *SD* = 5.34), ranging from 1 to 63 sessions, one year after the start of treatment.

*Community—complex* (Subsample 2, *n*_*2*_ = 143) provides referral-based treatment for more complex cases, often involving prior specialist care. These interventions focus on social engagement through work- or activity-based programmes, often in partnership with NAV and voluntary organisations. In this subsample, no clear number of sessions was registered, as they received longer interventions not documented in the same manner.

*Specialist mental health services* (Subsample 3, *n*_*3*_ = 2047) provide referral-based treatment for patients with moderate to severe conditions. Treatments typically involve eclectic psychotherapeutic approaches focused on reducing symptoms and improving daily functioning and quality of life [37]. Patients in specialist care received a median of 10 sessions (*M* = 12.75, *SD* = 10.44), ranging from 1 to 88 sessions. Of these sessions, 27% were categorised as assessments, 57% as psychotherapy, 7% as medical follow-up, 4% as support, and 5% as other consultations. About 65% of contacts were provided by psychologists, 21% by medical doctors, 6% by nurses, 3% by social workers, 1% by physiotherapists, and 4% by other healthcare professionals like support workers or occupational therapists. Information regarding possible medication use and somatic examinations was not reported. In this study, the content and type of treatment were not systematically recorded; however, by national guidelines, clinicians are required to adhere to evidence-based treatment methods. Manual-based treatments have become increasingly central to evidence-based treatment and mental health policy, driven by institutions like the American Psychological Association (APA) and the National Institute for Health and Care Excellence (NICE), which emphasise structured, guideline-driven interventions [38]. Despite this, routine practice still typically involves a variety of therapeutic non-manualised approaches [39].

### Variables

The primary outcome was changes in work disability, categorised at each time point or time intervals into five states: working, partial sick leave, full sick leave, WAA, and disability pension. Working was defined as not receiving any type of medical benefits. Change in work disability status (for example, moving from any form of medical benefit to partial or none, labelled working) was coded as an indicator of successful return-to-work or return to full-time studies for students. Those with partial disability pensions were considered actively employed through their work capacity. This study used seven distinct time points, each corresponding to a specific date relative to the start of treatment: 12 months pre-treatment, 6 months pre-treatment, 3 months pre-treatment, at the start of treatment, 3 months post-treatment, 6 months post-treatment, and 12 months post-treatment. Additionally, four broader time intervals were constructed to capture longer-term transitions: 12–6 months pre-treatment, 6–0 months pre-treatment, 0–6 months post-treatment, and 6–12 months post-treatment.

### Statistical methods

To visualise daily changes in work disability status, for each patient, we categorised each day into one of five benefit categories. The proportion of patients in each category was then summarised for each day pre- and post-treatment and presented using stacked bar charts, enabling a graphical representation of changes over time.

Three complementary analytical strategies were employed to examine changes in work disability status over time. First, McNemar’s test for paired nominal data was used in Stata 18 to assess within-subject changes in the distribution of individuals across work disability categories in the total sample at seven consecutive time points.

To explore differences in return-to-work outcomes across care levels, we compared patients receiving treatment in primary care settings (Community—mild to moderate) with those in Specialist mental health services.

Second, a longitudinal mixed-effects regression analysis was conducted in Stata 18 to examine the differences in absenteeism between care levels (Community—mild to moderate and Specialist mental health services) across the four time intervals (12–6 months pre-treatment, 6–0 months pre-treatment, 0–6 months post-treatment, and 6–12 months post-treatment). Absenteeism was defined as the number of days per six-month interval where the individual was on either partial sick leave, full sick leave, WAA, or disability pension. A random intercept for individual ID was included in the model. No covariates were included in this model, and results are presented as unadjusted group-level differences over time. There were no missing data since participants were included based on accepting participation at the start of treatment.

Third, to identify distinct patterns of work disability outcomes (working, partly sick leave, full sick leave, WAA, and disability pension) across the seven time points, sequence and cluster analysis were conducted using the TraMineR package in R (v. 4.3.1). The sequences were compared using Hamming distance as the dissimilarity measure and Ward’s hierarchical clustering algorithm to group individuals into clusters with similar trajectories. The optimal number of clusters (*k* = 5) was chosen based on interpretability and clinical relevance. Sequence analysis allows full preservation of individual transitions without assuming predefined trajectory shapes, making it particularly suited for capturing heterogeneous work disability patterns in this naturalistic cohort [40]. Finally, multinomial logistic regression analyses were performed in R to examine associations between cluster membership and participant characteristics, adjusting for age, sex, and clinical setting. Cluster 1 (Stable Work ability) served as the reference group. Clinic was dummy-coded, with Community—mild to moderate serving as the baseline. Odds ratios (ORs) and 95% confidence intervals were calculated to estimate the relative likelihood of assignment to Clusters 2, 3, 4, and 5 compared with Cluster 1. Bivariate differences across clusters were examined using ANOVA and chi-square tests.

## Results

### Participants

Of the 2,609 participants included, participation rates were 79% in Specialist mental health services and 74% in Community mental health services [41, 42]. No participants were lost to follow-up, as complete medical benefit data were retrieved from national registries. The mean age was 30.5 years (*SD* = 10.84), and 64% were female in the total sample. At treatment start, 62.3% were working, 7.8% were on partial sick leave, 11.5% on full sick leave, 11.8% on WAA, and 6.7% were receiving disability pension. On average, patients had received 91.6 days of medical benefits in the year before treatment (*SD* = 132.7; median = 13 days), with 43.5% reporting none. Additional demographic and baseline clinical characteristics are presented in Table 1.

**Table 1:** Demographic and clinical characteristics

Variables	Community mental health services (*n =* 562)		Specialist mental health services (*n*_3_ = 2047)	Total sample (*N =* 2609)
	Mild to moderate (*n*_1_ = 419)	Complex (*n*_2_ = 143)		
*Demographics at the start of treatment*				
Age, Mean (*SD*)	34.21 (11.39)	35.43 (13.49)	29.38 (10.24)	30.48 (10.84)
Female, *n* (%)	302 (72.08)	103 (72.03)	1273 (62.19)	1678 (64.32)
*Work disability status at the start of treatment*,* n (%)*				
Working	252 (60.14)	49 (34.27)	1323 (64.63)	1,624 (62.25)
Partly sick leave	74 (17.66)	5 (3.50)	124 (6.06)	203 (7.78)
Full Sick leave	60 (14.32)	4 (2.80)	236 (11.53)	300 (11.50)
WAA	11 (2.63)	39 (27.27)	258 (12.60)	308 (11.81)
Disability pension	22 (5.25)	46 (32.17)	106 (5.18)	174 (6.67)
Medical benefits* 1 year before the start of treatment, Days				
Mean *(SD)* Median	71.04 (106.19) 21	227.79 (165.37) 365	86.26 (129.75) 6	91.57 (132.70) 12
Medical benefits* 1 year before the start of treatment, n (%)				
No days	149 (35.56)	32 (22.38)	953 (46.56)	1134 (43.46)
1–90 days	161 (38.42)	18 (12.59)	496 (24.23)	675 (25.87)
90–180 days	55 (13.13)	5 (3.50)	188 (9.18)	248 (9.51)
181–270 days	177 (4.06)	6 (4.20)	88 (4.30)	111 (4.25)
>270 days	37 (8.83)	82 (57.34)	327 (15.97)	446 (17.09)

*Note: WAA* = Work Assessment Allowance; *Medical benefits = Partial and full sick leave, WAA, and disability pension

### Descriptive data: changes in diagnoses associated with work disability

**Fig. 1:**
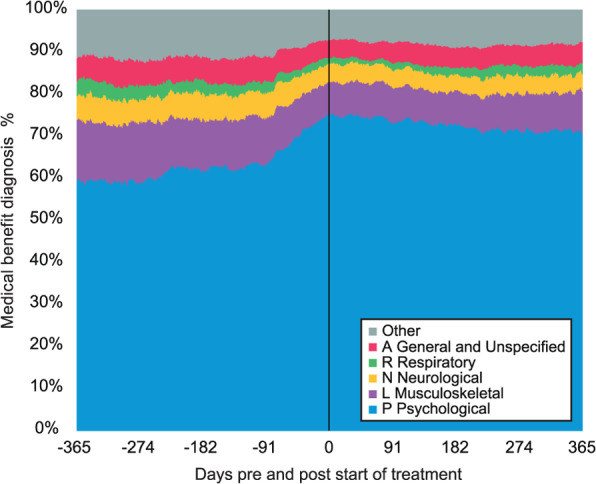
Changes in ICPC-2 diagnos is for work disability 1 year pre-and post-treatment in the total sample (*N =* 2609). This represents all registered medical benefits, with the sample size of diagnoses varying daily

One year before the start of treatment, psychological problems (ICPC-2: P) were the most common diagnoses, accounting for 54% (*n =* 311) of all medical benefit cases at that point (*n =* 580). Musculoskeletal conditions represented 13% (*n =* 76), neurological 5% (*n =* 29), general and unspecified 5% (*n =* 29), respiratory 3% (*n =* 18), and the combined categories of the other diagnoses accounted for 10% (*n =* 60). The latter refers to a custom group composed of different diagnoses, not indicating comorbidity. At the start of treatment, the proportion of P-diagnoses had increased to 70% (*n =* 740) of all medical benefit cases that day (*n =* 1050). One year after the start of treatment, P-diagnoses remained the most prevalent diagnostic category at 66% (*n =* 592) of all diagnoses that day (*n =* 903). The distribution of diagnostic categories over time is illustrated using a stacked bar chart in Fig. 1.

### Outcome data: changes in work disability

**Fig. 2:**
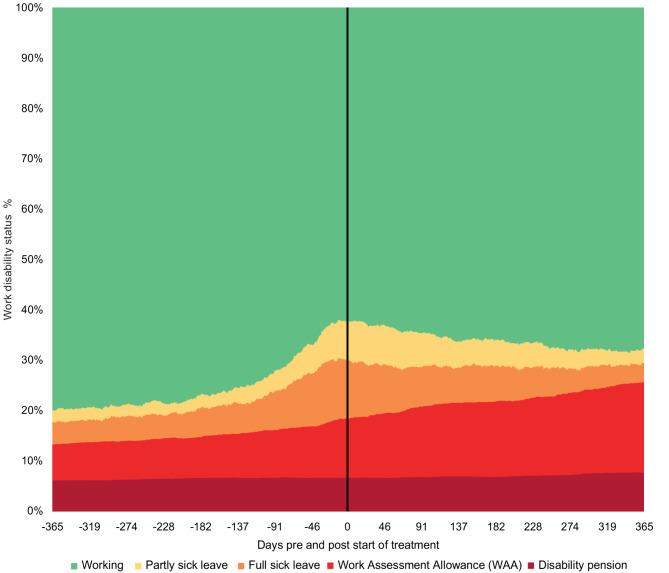
Changes in work disability 1 year pre-and post-treatment in the total sample (*N =* 2609)

Daily changes in medical benefits over time are shown in Fig. 2. Absenteeism, defined as the sum of any form of medical benefits, rose sharply from 20.1% one year before treatment to 37.8% at treatment start, representing a total 88.0% increase from one year before treatment. After treatment started, absenteeism decreased to 32.4% at the one-year follow-up, representing a 14.3% relative reduction from the treatment start. However, this still reflects a 61.1% increase compared to one-year pretreatment. WAA increased steadily over time, from 7.2% one year before treatment to 18.0% one year after treatment, representing a 152.3% increase. McNemar’s test identified a significant change (*p* <.001) in the proportions of individuals across different benefit states across seven time points. Detailed numerical values across seven time points are presented in Supplementary Table S1.

**Fig. 3:**
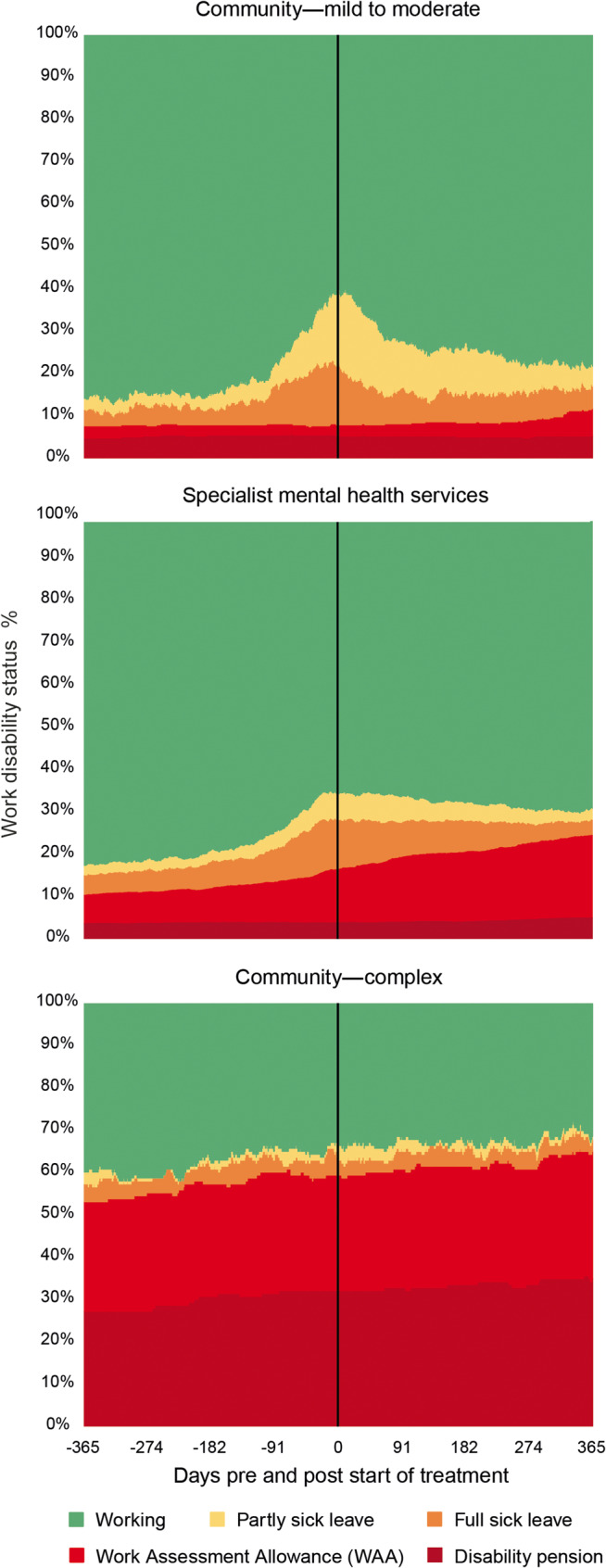
Changes in work disability in the three samples (Community—mild to /moderate, Specialist mental health services, and Community—complex)

Figure 3 presents the distribution of patients across work disability status for the three different clinical settings. As illustrated, the Community—complex had consistently higher levels of work disability over time. In contrast, Community—mild to moderate demonstrated a greater increase in work ability compared to Specialist mental health services.

### Main results: trajectories of work disability

**Fig. 4:**
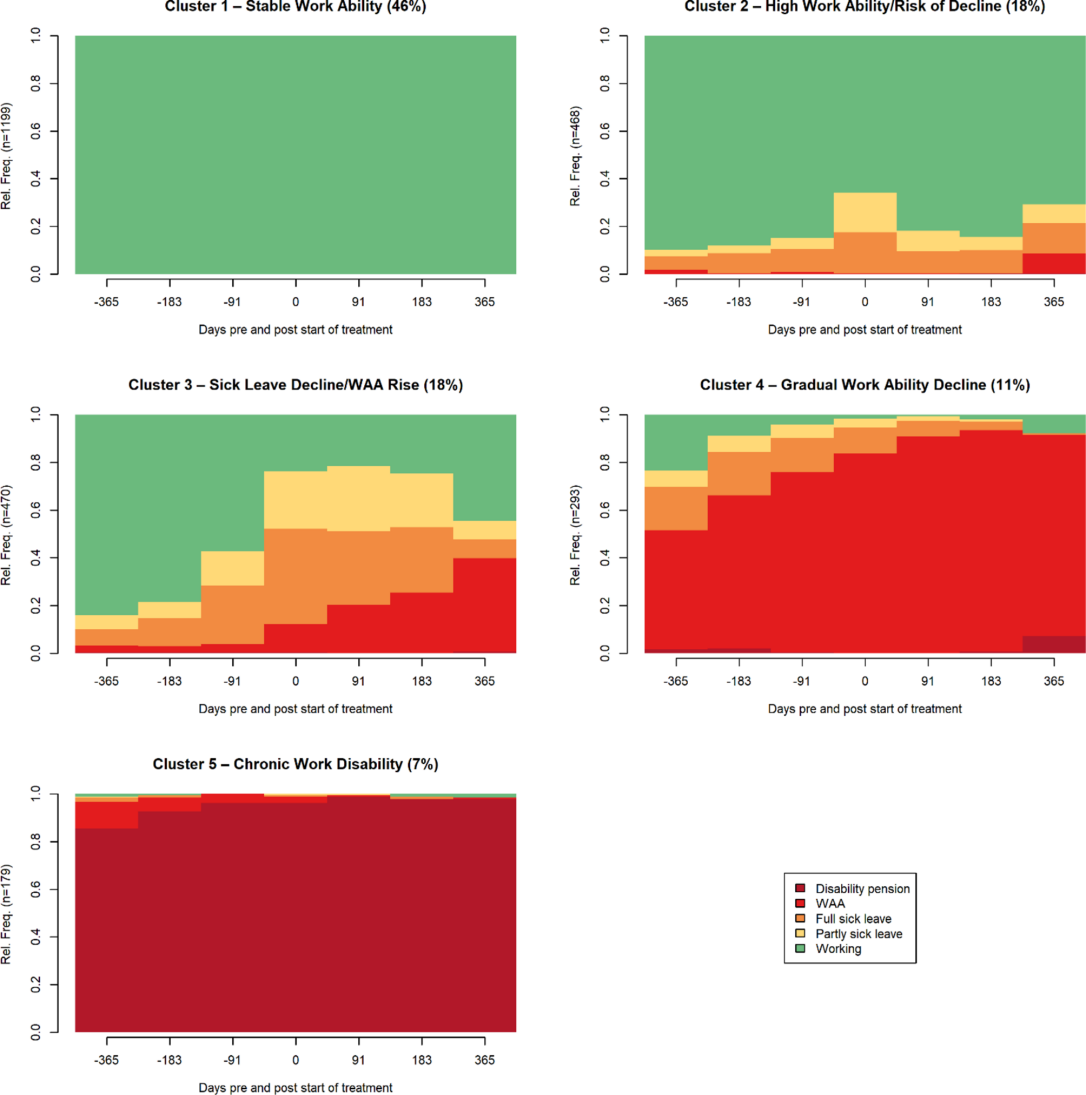
Work disability trajectories by cluster 1–5: Stable Work Ability to Chronic Work Disability, clustered from individual-level work disability status sequences (*N* = 2609). *Note*: Each trajectory represents the average work disability status across seven time points: 12, 6, and 3 months before treatment, at treatment start, and 3, 6, and 12 months after treatment. Five trajectory clusters illustrate distinct pre/post-treatment work disability patterns organised from most to least positive work ability outcome

A longitudinal mixed-effects regression analysis revealed a significant interaction between time and treatment setting (Community—mild to moderate and Specialist mental health services) (*χ²*(3) = 630.76, *p* <.001), indicating that absenteeism varied significantly across the four six-month intervals. There was also a significant main effect of clinical setting, with lower overall absenteeism observed in Community—mild to moderate compared to Specialist mental health services (*β* = −9.18, *z* = − 2.36, *p* =.018). Both groups showed an increase in absenteeism following treatment initiation (0–6 months post), but group trajectories diverged during the 6–12-month follow-up. Specifically, individuals in Specialist mental health services showed a steady increase in predicted absenteeism from 36.2 days (12–6 months pre-treatment) to 63.3 days (0–6 months post-treatment), followed by a slight decline to 58.7 days in the final interval (6–12 months post-treatment). In contrast, Community—mild to moderate began with 27.0 days of absenteeism, peaked at 54.1 days during the first six months after treatment, and then showed a more substantial reduction to 42.5 days in the final follow-up period. The interaction between time and group was significant in the 6–12 months post-treatment interval (*β* = −7.03, *z* = − 2.27, *p* =.023), indicating that the reduction in absenteeism was significantly greater in the Community—mild to moderate than in Specialist mental health services during long-term follow-up. To further explore patterns of work disability outcomes, a sequence and cluster analysis was conducted, identifying five distinct trajectory clusters over the seven time points. The clusters are organised by work ability outcomes, from most to least positive, see Fig. 4. The largest group, Cluster 1, “Stable Work Ability” (46%), comprised individuals who remained consistently in work throughout the study period. Cluster 2 (18%), “High Work Ability/Risk of Decline”, included individuals with generally high work ability and some sick leave, mainly showing a reduction in partial sick leave but a risk of increased WAA toward the end of the period. Cluster 3 (18%), “Sick Leave Decline/WAA Rise”, reflected a reduction in sick leave; however, a possible benefit transition with increased WAA. Cluster 4 (11%) labelled “Gradual Work Ability Decline” was characterised by a steady transition from work and short-term benefits to sustained WAA. Finally, Cluster 5, “Chronic Work Disability” (7%), consisted almost entirely of individuals on disability pension, with little or no work ability. Descriptive characteristics of each cluster are summarised in Table 2.

**Table 2:** Descriptive characteristics of the five identified work disability trajectory clusters (*N* = 2609)

Cluster	*n*	%	Age (M)	Female (%)	Community mental health services (%)		Specialist mental health services (%)
					Mild to moderate	Complex	
C1	1,199	46.0	25.5	63.9	14.5	2.5	83.0
C2	468	17.9	31.6	64.1	26.9	3.2	69.9
C3	470	18.0	34.6	64.3	18.1	2.8	79.1
C4	293	11.2	34.0	62.8	4.1	13.0	82.9
C5	179	6.9	44.2	70.4	12.3	26.3	61.5

*Note: C1* = Stable Work ability, *C2* = High work ability/Risk of Decline, *C3* = Sick Leave Decline/WAA Rise, *C4* = Gradual Work ability Decline, *C5* = Chronic Work Disability; The percentages in the “%” column refer to the proportion of individuals within each cluster out of the total sample. Percentages under clinical settings refer to the row-wise distribution within each cluster

A multinomial logistic regression with Cluster 1 as the reference category, was conducted to examine predictors of cluster membership. Clinical setting was a significant predictor. Compared to patients treated in Community—mild to moderate, those treated in Specialist mental health services had more than six times higher odds of belonging to the cluster 4 (*OR* = 6.31, *p* <.001), and over twice as high odds of belonging to cluster 5 (*OR* = 2.35, *p* =.002), and were less likely to belong to Cluster 2 than Community—mild to moderate (*OR* = 0.67, *p* =.005). Individuals treated in Community—complex had substantially higher odds of being in Cluster 4 (*OR* = 31.06, *p* <.001) or Cluster 5 (*OR* = 28.59, *p* <.001). Being female was associated with higher odds of belonging to Cluster 5 (OR = 1.70, *p* =.008), although sex was not significantly associated with other clusters (*χ*²(4) = 3.28, *p* =.51). Older age was a significant predictor of worse work ability outcomes across all clusters (*p* <.001). Each additional year of age increased the odds of belonging to Cluster 5 (Chronic Work Disability). Participants in Cluster 5 were significantly older than those in Cluster 1 (OR = 1.20, *p* <.001). Detailed results of the Multinomial Logistic Regression Predicting Cluster Membership are presented in Supplementary Table S2.

## Discussion

Work disability due to mental disorders is on the rise across many high-income countries, yet little is known about changes in work disability associated with routine mental health care [4, 43]. To our knowledge, this is the first study to detail the trajectory of work disability before and after the start of routine mental health treatment. In this large cohort, based on national registry data, we found that nearly half of the patients maintained stable work ability throughout the year before and after treatment. However, we also found that although absenteeism declined modestly after treatment, levels of long-term medical benefits continued to rise, and as hypothesised, a substantial proportion of patients transitioned into more permanent absenteeism. These findings suggest that routine mental health care, as it is currently delivered, may have a limited impact on preventing long-term work disability. Crucially, this study shows that for many patients, starting treatment does not mark the beginning of functional recovery, but rather a persistent or even a gradual deterioration of work ability. In line with a previous study, these results imply a need for timely interventions and preventive measures to mitigate long-term negative outcomes [32]. Understanding the real-world effects of mental health treatment on work disability is therefore an urgent priority for research and policy.

Tracking work disability over two years revealed a sharp increase in absenteeism the year before treatment, peaking at 38% at the start. Consistent with our hypothesis based on previous studies [31, 32], this study showed an inverted U-shaped curve in sick leave, with an initial increase followed by a 22% relative reduction (from 19 to 15%) after six months. This curve aligns with previous research [28, 31, 32] identifying a reduction in sick leave at follow-up, though the overall reduction of absenteeism in this study was modest (14%) after one year, with no clear downward trend. Critically, while short-term sick leave decreased, long-term medical benefits continued to increase throughout the years. This rise in long-term benefits counteracts the reduction in short-term sick leave, resulting in a maintenance of a relatively stable level of work disability, regardless of the level of care. This post-treatment “plateau” in absenteeism is concerning, as WAA has been termed a “waiting list for disability pension” [5]. Given the strong association between prolonged sick leave and future disability pension, and the fact that most individuals remain on disability pension until retirement or death [44, 45], this trend underscores substantial challenges in the welfare system’s ability to facilitate timely work ability [46].

Sequence analysis identified five work disability trajectories, where approximately 30% of patients, corresponding to clusters 3 “Sick Leave Decline/WAA Rise” and 4 “Gradual Work Ability Decline”, experienced reduced sick leave followed by a benefit transition to long-term medical benefits, while 7% maintained chronic work disability. These findings challenge the assumption that symptom improvement following treatment naturally leads to increased work ability [13]. Although routine care in Nordic outpatient settings has been associated with symptom improvements [47, 48], many patients remained on medical benefits post-treatment, emphasising the need to explicitly target work disability as a primary treatment objective, rather than assuming an implicit outcome.

Several factors were associated with worse work disability. Older age was found to be a negative prognostic indicator for the development of chronic work disability, with a 20% increase in odds for each additional year of age. Female sex was also linked to a higher risk of more permanent work disability, reinforcing previous findings that women are disproportionately affected by long-term absenteeism [49]. Furthermore, the treatment setting also played a critical role. Patients in Community—mild to moderate showed greater improvements in work ability than those treated in Specialist mental health services. Still, this difference in work disability could be expected, given the presumed differences in patient severity: patients in the Community—mild to moderate are likely to experience less severe challenges, facilitating faster return-to-work than patients in Specialist mental health services. However, in a previous study based on a similar Norwegian routine care sample, only small differences in symptoms and functional impairment were detected between these clinical settings [37]. This suggests that the treatment context and timing may be essential. While Community—mild to moderate offers a timely start of treatment (aiming to start treatment within two weeks after self-referral), Specialist mental health services require a referral from a GP and additionally an average waiting period of 57 days for assessment, followed by treatments [50]. Consequently, Community—mild to moderate likely access care earlier, even at a possible sub-clinical stage or soon after medical benefit began. Accordingly, this easy access to Community services may play a critical role in explaining differences in work disability, as timely treatment is key in facilitating earlier recovery, preventing symptom worsening, and progression to long-term benefits [51, 52]. Yet, the reasons behind these differences in work disability remain to be explored.

As expected, given the severity and complexity of their conditions, Community—complex consistently exhibited higher levels of work disability across the two years. This group represents individuals with substantial and multifaceted barriers to work participation, and interventions in this setting aim more broadly at social inclusion and gradual functional improvements rather than rapid return-to-work.

The steady increase in long-term medical benefits following treatment suggests that routine mental health services may be insufficient to address work disability. Integrating work-focused interventions into routine care, targeting both symptom reduction and work disability, is crucial, as emphasised by the OECD and World Health Organization [5, 51]. However, mental health services often prioritise symptom management over absenteeism, frequently leaving the management of work disability to welfare agencies such as the Norwegian Labour and Welfare Administration, once treatment ends [12, 36]. At the same time, focusing exclusively on work disability without addressing underlying mental health issues may extend absenteeism [20]. Comprehensive interventions addressing both symptom burden and work disability are therefore needed in order to enhance return-to-work [20].

Although this study provides important new insights into work disability outcomes following routine mental health care, more research is warranted, as few studies have systematically evaluated routine care settings [27]. Further research is needed to determine which treatment components and service models are most effective in enhancing return-to-work. However, given the limited improvements in work disability and the rise of long-term medical benefits observed, there is a growing need to rethink and adapt routine mental health services, especially in Specialist mental health services. Establishing the reduction of work disability as an explicit treatment goal, integrating work-focused approaches, and improving coordination between healthcare and welfare systems may be critical steps toward better supporting work ability. Importantly, such service adaptations should themselves be subject to empirical evaluation to ensure that they are both effective and accessible in routine care facilities. By establishing a baseline for the relationship between routine mental health treatment and work disability, this study provides a foundation for more targeted evaluations and the development of future intervention strategies aimed at improving work ability outcomes.

### Strengths and limitations

A key strength of this study is the use of comprehensive register data from NAV, covering all medically certified benefit days for a large sample of individuals receiving routine public mental health care, regardless of treatment level. Additionally, including all diagnostic groups helped reduce the underreporting of psychiatric diagnoses as reasons for work disability [7]. To our knowledge, this approach has not been previously employed, and given that many countries with similar healthcare systems face high and increasing sick leave rates [43], the findings of this study may hold international relevance.

However, some limitations need to be acknowledged. First, the prospective cohort design limits our ability to draw causal conclusions. Consequently, the effect of TAU on work disability compared to other active treatments or no treatment remains unknown. Secondly, the register-based data only included physician-certified medical benefits, excluding self-reported sick leave. As Norwegian employees are allowed at least three self-certified sick days four times annually, this likely led to an underestimation of absenteeism. Third, ethical constraints prevented data collection on non-participants, posing a risk of selection bias. Fourth, this study fails to provide detailed information on TAU characteristics. Fifth, the lack of a longitudinal follow-up beyond one year limits our ability to capture long-term outcomes, particularly potential increases in disability pension claims. Sixth, the registry only covered medical benefits, excluding individuals not receiving such support (e.g., unemployed). This limits our ability to distinguish between medically defined work disability and other forms of non-employment. However, comparable data showed low unemployment (2.7%), suggesting minimal impact on the results [53]. A seventh limitation is the lack of detailed information on treatment and patient groups, as the primary aim was to establish a baseline for future research on registered data examining long-term medical benefit trajectories and variations in routine care. Since this study is a part of a larger research project that includes self-reported measures of symptoms and functioning, future studies will address this by incorporating self-reported measures along with more detailed data.

## Conclusion

This study shows that while a substantial proportion of patients maintained stable work ability during the year before and after the start of routine mental health treatment, a growing number transitioned to long-term medical benefits following treatment. Although a modest reduction in sick leave was observed, overall benefit use was still high. Older age, female sex, and treatment in specialist care were associated with work disability. By establishing a baseline for work disability trajectories, this study highlights the urgent need to integrate work ability goals into routine mental health care to better prevent transitions to long-term work disability. Given the global rise of work disability [43], future studies should investigate how to improve work ability outcomes following routine care.

## Supplementary Information

Supplementary Material 1

## Data Availability

The data from the Norwegian National Social Security System Registry, managed by the Norwegian Labour and Welfare Administration (NAV), Nidaros Community Mental Health Centre, and Trondheim Municipality, are subject to restrictions. They are only available for use under license by researchers involved in the current study and are not publicly accessible.

## Abbreviations

APA: American Psychological Association
CBT: Cognitive Behavioural Therapy
GP: General Practitioner
ICPC-2: International Classification of Primary Care, 2nd Edition
NAV: Norwegian Labour and Welfare Administration
NICE: National Institute for Health and Care Excellence
NSD: Norwegian Centre for Research Data
OECD: Organisation for Economic Co-operation and Development
REK: Regional Committee for Medical and Health Research Ethics
SE: Standard Error
SD: Standard Deviation
WAA: Work Assessment Allowance (Temporary and Long-term medical benefit)
