# Publisher Correction: Work disability status following routine mental health treatment: a Norwegian registry-based cohort study

**DOI:** 10.1186/s12913-025-13389-y

**Published:** 2025-08-28

**Authors:** Jakob Lundqvist, Martin Schevik Lindberg, Martin Brattmyr, Audun Havnen, Lene Aasdahl, Stian Solem, Odin Hjemdal

**Affiliations:** 1 Department of Psychology, Norwegian University of Science and Technology (NTNU), Trondheim, Norway; 2 Health and Welfare, Trondheim Municipality, Trondheim, Norway; 3 Division of Psychiatry, Nidaros Community Mental Health Centre, St. Olavs University Hospital, Trondheim, Norway; 4 Department of Public Health and Nursing, Norwegian University of Science and Technology (NTNU), Trondheim, Norway; 5 Unicare Helsefort Rehabilitation Center, Rissa, Trondheim, Norway; 6 Diakonhjemmet Hospital, Oslo, Norway

**Correction: BMC Health Serv Res 25, 787 (2025)**

**https://doi.org/10.1186/s12913-025-12856-w**

In the online version of this article, Figure 3 was captured incorrectly due to a typesetting mistake (inversion of the colors).

The publisher apologizes to the authors and readers for the inconvenience caused by these errors.

