# Supplementary material for: Work disability status following routine mental health treatment: a Norwegian registry-based cohort study
Source: BMC Health Serv Res. 2025 Jun 2;25:787. doi: 10.1186/s12913-025-12856-w (PMC12128242; doi:10.1186/s12913-025-12856-w)
Supplement: Supplementary file 1 — Supplementary Material 1 [file 12913_2025_12856_MOESM3_ESM.pdf]

Supplementary

Supplementary Table S1. Changes in work disability 1 year pre-and post-treatment across care levels ($N = 2609$).

Work Disability Status	A: 12m pre *n (%)*	B: 6m pre *n (%)*	C: 3m pre *n (%)*	D: Start *n (%)*	E: 3m post *n (%)*	F:6m post *n (%)*	G: 12m post *n (%)*	Comp.	χ^2	*p*
Working	2085 (79.92)	2007 (76.93)	1878 (71.98)	1624 (62.25)	1686 (64.62)	1679 (64.35)	1765 (67.65)	A > B<br>B > C<br>C > D<br>D < E	20.84<br>57.58<br>150.74<br>12.73	< .001<br>< .001<br>< .001<br>< .001
Partly sick Leave	62 (2.38)	66 (2.53)	105 (4.02)	203 (7.78)	175 (6.71)	182 (6.98)	74 (2.84)	B < C<br>C < D<br>E > F<br>F > G	13.70<br>47.08<br>10.53<br>20.79	< .001<br>< .001<br>.001<br>< .001
Full sick leave	115 (4.41)	150 (5.75)	203 (7.78)	300 (11.50)	206 (7.90)	209 (8.01)	99 (3.79)	B < C<br>C < D<br>D > E<br>F > G	12.16<br>30.65<br>30.76<br>30.56	< .001<br>< .001<br>< .001<br>< .001
WAA	187 (7.17)	212 (8.13)	248 (9.51)	308 (11.81)	364 (13.95)	361 (13.84)	470 (18.01)	B < C<br>C < D<br>D < E<br>E < F<br>F < G	25.92<br>37.50<br>39.20<br>13.52<br>31.36	< .001<br>< .001<br>< .001<br>< .001<br>< .001
Disability pension	160 (6.13)	174 (6.67)	175 (6.71)	174 (6.67)	178 (6.82)	178 (6.82)	201 (7.70)	A < B<br>F < G	12.25<br>18.62	< .001<br>< .001

Note: WAA = Work Assessment Allowance; A–G represent time points from one year before to one year after the start of treatment (D); A = 12 months pre-treatment; B = 6 months pre; C = 3 months pre; D = treatment start; E = 3 months post; F = 6 months post; G = 12 months post; Only significant differences ($p < .05$) are reported; non-significant results are omitted for clarity.

Supplementary Table S2. Multinomial logistic regression predicting cluster membership ($N = 2609$)

Predictor	Comparison (C1)	OR	95% CI	*SE*	*p*
Age at start	Cluster 2 vs 1	1.09	(1.07, 1.11)	0.007	< .001
Age at start	Cluster 3 vs 1	1.12	(1.11, 1.14)	0.007	< .001
Age at start	Cluster 4 vs 1	1.13	(1.11, 1.14)	0.008	< .001
Age at start	Cluster 5 vs 1	1.20	(1.18, 1.21)	0.009	< .001
Sex (Female)	Cluster 2 vs 1	1.09	(0.87, 1.36)	0.119	0.460
Sex (Female)	Cluster 3 vs 1	1.22	(0.96, 1.54)	0.122	0.108
Sex (Female)	Cluster 4 vs 1	1.18	(0.89, 1.56)	0.144	0.258
Sex (Female)	Cluster 5 vs 1	1.70	(1.15, 2.49)	0.201	0.008
Community–complex	Cluster 2 vs 1	0.98	(0.50, 1.95)	0.345	0.962
Community–complex	Cluster 3 vs 1	1.49	(0.72, 3.10)	0.373	0.283
Community–complex	Cluster 4 vs 1	31.06	(14.27, 67.61)	0.403	< .001
Community–complex	Cluster 5 vs 1	28.59	(13.78, 59.33)	0.384	< .001
Specialist services	Cluster 2 vs 1	0.67	(0.51, 0.88)	0.143	0.005
Specialist services	Cluster 3 vs 1	1.36	(0.99, 1.85)	0.162	0.058
Specialist services	Cluster 4 vs 1	6.31	(3.39, 11.76)	0.317	< .001
Specialist services	Cluster 5 vs 1	2.35	(1.38, 4.00)	0.279	0.002

Note: OR = odds ratio; *SE* = standard error; 95% CI = 95% confidence interval; C1 = Stable Work ability; C2 = High work ability/Risk of Decline; C3 = Sick Leave Decline/WAA Rise; C4 = Gradual Work ability Decline; C5 = Chronic Work Disability; Reference group for multinomial logistic regression is Cluster 1 (Stable Work ability); Clinical setting reference: Community—mild to moderate. Sex is coded 0 = male, 1 = female.
